# Roles of TRP Channels in Neurological Diseases

**DOI:** 10.1155/2020/7289194

**Published:** 2020-09-04

**Authors:** Rui Wang, Sheng Tu, Jianmin Zhang, Anwen Shao

**Affiliations:** ^1^Department of Neurosurgery, The Second Affiliated Hospital, School of Medicine, Zhejiang University, Hangzhou, China; ^2^State Key Laboratory for Diagnosis and Treatment of Infectious Diseases, Collaborative Innovation Center for Diagnosis and Treatment of Infectious Diseases, The First Affiliated Hospital, College of Medicine, Zhejiang University, Hangzhou, China

## Abstract

Transient receptor potential (TRP) proteins consist of a superfamily of cation channels that have been involved in diverse physiological processes in the brain as well as in the pathogenesis of neurological disease. TRP channels are widely expressed in the brain, including neurons and glial cells, as well as in the cerebral vascular endothelium and smooth muscle. Members of this channel superfamily show a wide variety of mechanisms ranging from ligand binding to voltage, physical, and chemical stimuli, implying the promising therapeutic potential of TRP in neurological diseases. In this review, we focus on the physiological functions of TRP channels in the brain and the pathological roles in neurological disorders to explore future potential neuroprotective strategies.

## 1. Introduction

The transient receptor potential (TRP) channels are a superfamily of cation channels that play critical roles in the responses to diverse environmental changes and stimuli. Mutations in TRP were originally related to an abnormal behavioral phenotype in response to light and characterized by an electroretinogram phenotype in *Drosophila*. The mutant strain of *D. melanogaster* showed a transient receptor potential to constant light rather than the continuous response recorded in the wild type, which leads to the origin of the name TRP [1]. The TRP channel superfamily is conserved in yeast, invertebrates, and vertebrates. On the basis of sequence homology, this superfamily can be subdivided into 7 subfamilies including TRPC (canonical), TRPV (vanilloid), TRPM (melastatin), TRPP (polycystin), TRPML (mucolipin), TRPA (ankyrin), and TRPN (NOMPC-like); TRPN exists only in invertebrates and fish, but not in mammals. TRP channels are widely expressed in multiple human organs, such as the brain, heart, kidneys, and lungs. TRP channels largely demonstrate permeability to both monovalent and divalent cations, as well as trace metal ions. Interestingly, they can be activated by a variety of gating mechanisms, including ligand binding, voltage and changes in temperature, and covalent modifications of nucleophilic residues [2]. Given the widespread distribution and complex cellular sensors of TRP channels, it is not surprising that TRP channels participate in the pathogenesis of diseases affecting different organ systems. In this review, we summarize the current knowledge on the pathological significance of TRP channels in the brain and give an outlook on potentially promising therapeutics targeting TRP.

## 2. Structure, Expression, and Function of TRP Channels in the Brain

### 2.1. Structure of TRP Channels

**Figure 1:**
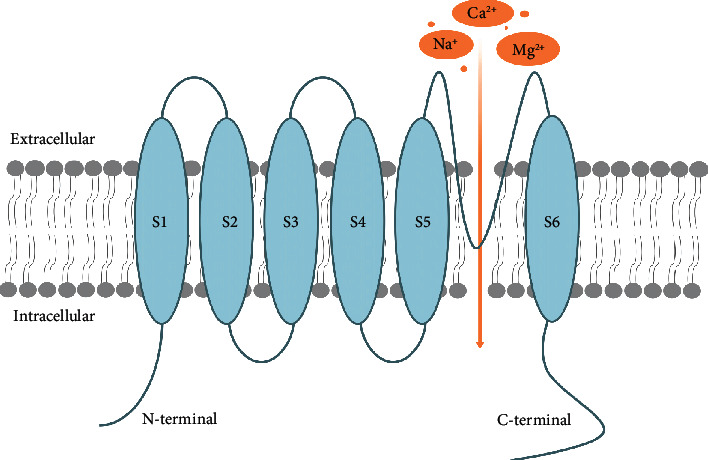
Schematic representation of TRP channels. TRP channels consist of 6 transmembrane domains and variable intracellular N- and C-termini, with a reentry loop that forms a pore located between S5 and S6. As cation channels, this structure constitutes an influx pathway mostly for Ca^2+^, Na^+^, and Mg^2+^.

TRPs form functional channels as either homotetramers or heterotetramers within the confines of the same or different subfamilies [3]. A typical TRP protein shares a common topology of six transmembrane helical segments (S1–S6), an intracellular amino (N), and a carboxy (C) terminal extension (Figure 1). The transmembrane domain can be divided in two building blocks: the voltage-sensing block formed by S1–S4 and the pore formed by S5 and S6. The pore forms an ion-conducting hole, which is hypothesized to shape the selectivity filter in TRP channels. The sensor block perceives the stimuli, transmits information to the gate, and causes a conformational change [4]. The transmembrane domain shares greater homology within a particular subfamily, and amino acid sequences of the pore block are the most strongly conserved across the TRP channels, which highlights the significance of the core channel structure. Intracellular N- and C-termini have less homology between subfamilies, and they are variable in length and sequence with a variety of domains and motifs. These diverse cytosolic domains play a role in channel assembly, activation, and regulation, which is one of the most fascinating structural aspects of TRP channels [5].

### 2.2. Expression and Functions of TRP Channels in the Brain

It is widely thought that TRPs play a significant role in the brain, considering their widespread expression and physiological functions. The activation of TRP channels by various stimuli and ligand binding contributes to changes in several monovalent and divalent cations, especially calcium. These contributions are important for several physiological functions, including sensitivity to stimuli (i.e., pheromone signaling cascades, thermosensation, and mechanosensation), ion homeostasis (i.e., calcium and magnesium reabsorption and osmoregulation), and motility (i.e., muscle contraction and vasomotor control) [2].

#### 2.2.1. TRP Channels in Neurons

The TRPC subfamily in mammals is the closest homolog to the *Drosophila* TRP. There are six mammalian TRPC proteins (TRPC1–6) widely distributed in the brain throughout embryogenesis to adulthood [6]. Extensive evidence has been provided for a critical role of TRPC1 in store-operated Ca^2+^ entry by interacting with stromal interaction molecule and Orai [7]. TRPC1 is involved in glutamate release, growth cone turning, and neurite outgrowth. TRPC2 is a pseudogene in human. As it is abundantly expressed in Purkinje cells, TRPC3 mediates metabotropic glutamate receptor- (mGluR-) dependent synaptic transmission [8]. TRPC4 and TRPC5 have an inhibitory role in neurite growth and morphogenesis and are thereby important for proper and balanced development of the brain [9, 10]. Together with TRPC3, TRPC6 plays an essential role in brain-derived neurotrophic factor- (BDNF-) induced axon guidance and neuron survival via Ca^2+^ signaling activation [11]. Overexpression of TRPC6 increases the number of spines in hippocampal neurons, leading to better spatial learning and memory. Thus, TRPC6 plays a role in synaptic and behavioral plasticity [12].

With widespread distribution in neurons throughout the brain, TRPV1 plays an important and versatile “nonsensory role” in neuronal activity and synaptic plasticity [13]. In the hippocampus, long-term depression (LTD) at excitatory synapses is induced by TRPV1 activation, and long-term potentiation (LTP) is reduced in Trpv1 and Trpv3 knockout mice [14, 15]. Furthermore, hippocampal endogenous cannabinoids such as the TRPV1 ligand anandamide could even reverse cognitive and learning impairments caused by chronic stress [16]. Moreover, this influence on synaptic plasticity is also found in the nucleus accumbens and dentate gyrus [17, 18]. TRPV2 expression is detected in the developing sensory and motor neurons, where it regulates axon outgrowth after activation by membrane stretch [19]. Additionally, TRPV2 is reportedly expressed in cultured hippocampal neurons and colocalizes with TRPV1 in a rat cortex, which may increase the functional diversity of this receptor family [20]. The thermosensor protein TRPV4 is activated at the physiological temperature in hippocampal neurons and regulates neuronal excitability and behaviors *in vitro* [21]. Unlike the other TRPs, TRPV5 and TRPV6 are the only highly selective Ca^2+^ channels and are rarely expressed in the brain.

TRPM2, formerly known as LTRPC2 or TRPC7, is a redox-sensitive TRP channel and is expressed in neurons of the hippocampus and substantia nigra [22, 23]. In particular, it is involved in the response to oxidative stress mediated by reactive oxygen species (ROS) and is associated with spontaneous firing rate, burst activity, and neuronal death [24, 25]. In addition, Trpm2 knockout mice show active axonal growth and impaired LTD, implicating a role in neuronal development and synaptic plasticity [22]. TRPM3 channel, which is abundantly expressed in hippocampus and cerebellar neurons, is activated by pregnenolone sulfate to modulate glutamatergic transmission [26]. TRPM4 and TRPM5 are Ca^2+^-impermeable monovalent channels, but they can be activated by intracellular Ca^2+^ to regulate inspiratory burst activity in the inspiratory neurons of the neonatal murine brainstem [27]. Primarily expressed in the small intestine and kidney, TRPM6 function in the nervous system is unclear now. TRPM7 is detected in the hippocampus, where it has a role in neuroprotection [28].

The function of TRPML in neurons is not completely known. TRPML1 is widely localized in the lysosome as the principle Ca^2+^ channel, where TRPML2-3 are involved in TRPML1 distribution and trafficking [29]. Disruption of the TRPML1 channel leads to a neurodegenerative disorder, which can be rescued by expression of TRMPL in neurons, glia, or hematopoietic cells [30].

The TRPA subfamily consists of only one mammalian member, TRPA1, which is expressed in the dorsal root, trigeminal ganglion neurons, and hair cells. It is known as a sensor for environmental stimuli and endogenous noxious substances.

#### 2.2.2. TRP Channels in Glial Cells

TRP expression has been detected in glial cells, though most of their functions remain unelucidated and under investigation.

In astrocytes, which are the most abundant cells in the brain, several isoforms of TRP are expressed. TRPC1-6 have been detected in cultured astrocytes with quantitative differences [31]. In fact, knockdown of the TRPC6 gene markedly reduced receptor-operated Ca^2+^ entry [32]. Besides, TRPC1 and TRPC3 are likely involved in store-operated Ca^2+^ entry, suggesting an important role for these proteins in the activation of excitable astrocytes [33]. TRPV1 is localized in the plasma membrane of astrocytes to detect bloodborne molecules in the sensory circumventricular organs of adult mouse brains [34]. TRPV2 expression is detected in plasma membrane and activated by very high temperature and endogenous lysophosphatidylcholine, the latter of which suggests that astrocytic TRPV2 might regulate neuronal activities in response to lipid metabolism [35]. TRPV4 activation in astrocytes is related to control of cell volume alongside aquaporin-4, amplification of neurovascular coupling responses via Ca^2+^-induced Ca^2+^ release, and regulation of neuronal excitability by release of gliotransmitters [36–38]. TRPM2 could be activated and upregulated by oxidative stress to induce a neuroinflammatory response [39]. Silencing TRPM7 decreases intracellular basal Mg^2+^ concentration without affecting Ca^2+^ concentration, leading to impaired proliferation and migration of astrocytes [40].

In microglia cells, the resident immune cells of the brain, only a few studies have been conducted about TRPs. TRPC3 in activated microglia contributes to the maintenance of BDNF-induced sustained intracellular Ca^2+^ elevation to suppress NO production [41]. Additionally, TRPV1 activation contributes to microglial migration and cell death via Ca^2+^-mediated mitochondrial damage [42, 43]. Furthermore, downregulation of TRPV1 and TRPV2 induced by cannabidiol enhances microglial phagocytosis [44]. Stimulation of TRPV4 suppresses abnormal activation of microglia by attenuating the driving force for extracellular Ca^2+^ [45]. TRPM2-mediated Ca^2+^ signaling in microglia contributes to neuronal degeneration via NO, as well as microglial migration and invasion during anti-inflammatory states [46, 47].

Single-cell transcriptomic data suggests that TRPC, TRPV and TRPM channels can be found in mouse oligodendrocyte lineages, though their functions, to date, largely remain unknown [48]. Among them, TRPC1-mediated Ca^2+^ influx is known to be involved in the proliferation of oligodendrocyte precursor cells by the golli products of the myelin basic protein gene [49].

In ependymal cells, TRPV1, TRPV3, and TRPA1 are found in the developing rat brain. TRP channel expression is found to be higher in choroid plexus than the ventricular lining ependyma. At the 19th day of gestation, all the expression is found to be decreased, which suggests different functions of regulating cerebral spinal fluid during development. Changes in TRP expression in gestation coincide with a period of ventricular shrinkage, which implies a possible role in the need to make space for the growing brain structure [50].

#### 2.2.3. TRP Channels in the Cerebral Blood Vessels

It has been reported that many TRP channels play functional roles in the cerebral blood vessels, including vascular endothelium and smooth muscle. TRPV3 is expressed in the endothelium of cerebral arteries, and its activation elicits vasodilation with a concurrent decrease of intracellular Ca^2+^ in arterial myocytes and smooth muscle hyperpolarization [51]. TRPA1 is specifically localized to the endothelial cell membrane projections proximal to smooth muscle layer. TRPA1-mediated Ca^2+^ influx elicits vasodilation involving endothelial cell Ca^2+^-activated K^+^ channels, thus inwardly rectifying K^+^ channels in arterial myocytes [52]. It is reported that TRPV4 and TRPM4 are detected differently in endothelial cells and smooth muscle cells, thus contributing to myogenic vasoconstriction of cerebral arteries via a Ca^2+^ influx induced by various factors [53, 54].

## 3. Roles of TRP Channels in Neurological Diseases

Considering the widespread expression and diverse functions of TRP channels in the brain, it is conceivable that dysfunction of these channels can have a profound effect on various pathological events of neurological and psychiatric disorders in animals (Table 1) and humans (Table 2) and their pharmacological interventions targeting TRP channels for corresponding diseases (Table 3).

### 3.1. Stroke

#### 3.1.1. Ischemic Stroke

In ischemic stroke, which is caused by the blockage of a cerebral blood vessel, excitotoxicity induced by Ca^2+^ overload is considered to be the most important mechanism of cell death. There is some accumulating evidence that TRPC6 expressed in neurons can protect from excitotoxicity after ischemia by suppressing intracellular Ca^2+^ elevation induced by N-methyl-D-aspartate (NMDA). It has been reported that downregulation or inhibition of TRPC6 during ischemia contributes to brain damage in rodent models, and upregulation or activation by hyperforin and resveratrol attenuates this damage [55, 56]. For example, in a transient model of middle cerebral artery occlusion (tMCAO), TRPC6 protein levels in neurons are found to be greatly reduced in ischemia as a result of NMDA receptor-dependent calpain proteolysis. This downregulation precedes and is responsible for ischemic neuronal cell death. Inhibiting TRPC6 degradation prevents ischemic brain damage, with a reduction in infarct volume at 24 hours after reperfusion, an improvement in behavior performance, and a lower mortality within 35 days after ischemia [57]. Thus, TRPC6 expression in neurons plays a beneficial role for neuronal survival and can be a potential therapeutic target after ischemic stroke. Additionally, an increase of TRPC4 protein is found in the striatum and hippocampus from 12 hours to 3 days after tMCAO operation, which suggests a role for this protein in acute and delayed neuronal injury after focal ischemia [59]. However, the role of TRPC4 in a positive or negative manner is still unknown after ischemia.

Studies have shown that TRPM2 and TRPM7 gating lies downstream of several signaling pathways in response to oxidative stress induced by cerebral ischemia and reperfusion injury, which is considered to be an important event leading to neuronal death. TRPM2 is considered a connection point that mediates Ca^2+^ overload in response to ROS. This sensitivity to ROS is attributable to the production of nicotinamide adenine dinucleotide and its metabolites such as adenosine diphosphoribose (ADPR) [60]. The inhibitors and RNA interference targeting TRPM2 efficiently suppress Ca^2+^ influx and ROS-induced neuronal death in cultured neurons or HEK cells. Using an in vivo stroke model, Trpm2 knockout mice exhibit smaller infarct volume at 48 hours after tMCAO operation when compared with wild-type mice. In hippocampal slices, sublethal concentrations of H_2_O_2_ increase baseline synaptic excitability in Trpm2 knockout but not wild-type neurons. This difference depends on the changed expression ratio of NMDA receptor subunits induced by the absence of TRPM2, which may then selectively upregulate survival signals and provide neuroprotection against ischemic cell death [61]. Interestingly, a TRPM2 inhibitor, tat-M2NX, provides protection from ischemic stroke with a smaller infarct volume in aged male mice but not female, suggesting a sexually dimorphic mechanism [62].

In addition to being expressed in neurons, several studies have highlighted the link between consequent injury and TRPM2 activation in nonneuron cells. The upregulation of TRPM2 has been observed to correlate with microglial activation from 1 to 4 weeks in a tMCAO stroke model, and the functional expression of a TRPM2-like conductance is confirmed in cultured microglia. Patch-clamp recordings from microglia demonstrate that increased intracellular ADPR or extracellular H_2_O_2_ induces an inward current, accompanying with activation of TRPM2 [63]. Besides, the TRPM2-mediated Ca^2+^ influx induces the production of proinflammatory chemokines in monocytes to aggravate inflammation [64]. Consistent with these findings, TRPM2 deficiency attenuates migratory capacities of neutrophils and macrophages into an ischemic brain thereby secondarily perpetuating brain injury [65]. Taken together, the above results indicate that TRPM2-mediated Ca^2+^ influx may be activated by intracellular messengers such as ADPR in response to oxidative stress after stroke, which may result in neuronal death and detrimental inflammation.

TRPM7 and TRPM2 channels share similar property of modulation by oxidative stress. RNA interference targeting TRPM7 also inhibits TRPM2 mRNA in primary cortical neurons, suggesting that the expression of the two proteins is interdependent [66]. TRPM7 activity is greatly enhanced after oxygen glucose deprivation, and TRPM7 inhibition blocks TRPM7 currents, anoxic Ca^2+^ uptake, ROS production, and anoxic death. This process is a pH-dependent channel potentiation resulting from an acidic environment due to ischemic stroke [67]. TRPM7 inhibitor carvacrol protects the brain from neonatal hypoxic-ischemic injury by reducing infarct volume, inhibiting apoptosis, and improving behavioral outcomes [68].

Moreover, TRPM4 is upregulated in vascular endothelium within the penumbra region after tMCAO. Blocking TRPM4 with 9-phenanthrol promotes tube formation on matrigel and improves vascular integrity after oxygen/glucose deprivation *in vitro* [69].

TRPVs play a complex role in cerebral ischemia. Trpv1 knockout mice show a lower neurological and motor deficits and infarct volume than wild-type mice in a tMCAO model. Furthermore, intracerebroventricular injection of capsazepine, a TRPV1 antagonist, leads to a reduction in infarct size and behavior deficits [70]. However, another study shows a contradictory role of TRPV1 in stroke. Hypothermia via a TRPV1 agonist, dihydrocapsaicin, provides neuroprotection following focal cerebral ischemia [71]. These findings indicate a promising but intricate target for ischemic stroke.

Besides, involvement of TRPV4 is found in both neuronal and glial pathophysiology associated with ischemia. In a hypoxic/ischemic model induced by a bilateral carotid occlusion combined with hypoxic conditions, TRPV4 expression was decreased in hippocampal pyramidal neurons with ongoing neuronal cell death, as well as an increase in reactive astrocytes with progression of reactive gliosis, suggesting a role for TRPV4 in neuronal loss and reactive gliosis following ischemic insult [72]. TRPV4 antagonism reduced the infarct size while its activation had an opposite effect in the tMCAO model [73]. Furthermore, there is increasing evidence to show that TRPV4 activation in astrocytes during ischemia results in a calcium influx into astrocytes and extracellular accumulation of glutamate, which causes Ca^2+^ overload of neurons and can trigger neuronal death [74, 75].

#### 3.1.2. Hemorrhagic Stroke (Intracerebral Hemorrhage and Subarachnoid Hemorrhage)

In hemorrhagic stroke caused by the rupture of a cerebral blood vessel, the blood-derived factors can extravasate into the brain parenchyma to participate in the pathophysiology of brain injury. It has been reported that TRPC3 can be dynamically upregulated by thrombin in rat primary cortical astrocytes. Additionally, it has been shown that this process contributes to the pathological activation of astrocytes through a feedforward upregulation of its own expression [76]. Consistently, in an intracerebral hemorrhage model induced by an intracerebral infusion of collagenase or autologous blood, TRPC3 inhibition by Pyr3 is found to reduce the perihematomal accumulation of astrocytes and ameliorate brain injury [77]. The TRPV4 antagonist, HC-067047, ameliorates neurological symptoms, brain edema and neuronal death, and preserves the blood-brain barrier after intracerebral hemorrhage, while its activation leads to the disturbance of Ca^2+^ homeostasis [78].

By contrast, decreased expression of TRPC3, TRPC5, and TRPM6 is found in cerebral vascular tissue from patients after hypertensive intracerebral hemorrhage through unknown mechanisms. Among them, TRPC3 mRNA correlates well with expression of hypoxia inducible factor-1a, suggesting its association with hypertension and hypoxic conditions [79]. Of note, after subarachnoid hemorrhage, TRPC1 and TRPC4 proteins are upregulated in canine smooth muscle, and the increased Ca^2+^ influx through TRPC channels mediates endothelin-1-evoked vasospasm [80]. Even after evaluating complex pathogenic functions, the role of TRP channels in hemorrhage still remains largely unknown.

### 3.2. Traumatic Brain Injury and Spinal Cord Injury

Traumatic brain injury (TBI) and spinal cord injury (SCI) can cause direct, immediate mechanical damage to tissue and indirect, delayed secondary damage that may continue from days to weeks. Recent studies have implicated TRPM channels in the pathophysiological processes of TBI and SCI. In an impact-acceleration model of diffuse TBI in adult male rats, TRPM2 mRNA and protein expression significantly increased in the cerebral cortex and hippocampus, susceptible regions to significant damage, following TBI, suggesting a role for TRPM2 in TBI [81]. It is also reported that TRPM2 may participate in TBI by regulating oxidative stress, apoptosis, and Ca^2+^ entry in a rat hippocampus by melatonin [82]. Additionally, after SCI, TRPM4 mRNA and protein were found to be upregulated in the capillaries and contribute to their fragmentation and formation of petechial hemorrhages [83].

In addition to Ca^2+^ overload, depletion of intracellular Mg^2+^ is associated with poor neurological outcomes after TBI. Specifically, TRPM6 and TRPM7 have been considered to potentially play a role in Mg^2+^ homeostasis as a result of neuronal injury, which is a topic that requires further study [84].

### 3.3. Brain Tumors

Glioma accounts for the majority of primary malignant tumors in the brain and is associated with poor prognosis. Recent studies have described the involvement of TRPCs and TRPVs in the regulation of malignant cell growth and progression. Consistent expression of TRPC1, TRPC3, TRPC5, and TRPC6 has been found in glioma cell lines and acute patient-derived tissues, which gives rise to small, nonvoltage-dependent cation currents and contributes to the resting conductance of glioma cells. Chronic inhibition of TRPCs by SKF96365 inhibits cytokinesis and results in multinucleated and enlarged cells that are histopathological hallmarks for glioblastoma multiforme, suggesting that a defect in TRPC channels may contribute to cellular abnormalities in this tumor type [85]. Similar morphological changes have been associated with high invasiveness observed in human glioma cells, which is induced by Ca^2+^ entry via TRPC1 and activated Cl^−^ currents [86]. Moreover, glioma cell proliferation is inhibited by cannabidiol-induced TRPV2 activation, resulting from TRPV2-dependent Ca^2+^ influx and increase in the uptake of cancer chemotherapeutic drug, which, in parallel, potentiates cytotoxic activity in human glioma cells [87].

Human TRPML2 is found both in normal astrocytes and in neural stem/progenitor cells, as well as in glioma tissues of different levels and high-grade glioma cell lines of astrocytic origin. Knockdown of TRPML2 inhibits viability and proliferation and triggers apoptosis of glioma cell lines [88]. Therefore, TRP channels are potential and promising targets that interfere with relentless glioma growth and invasion.

### 3.4. Neurodegenerative Diseases

#### 3.4.1. Alzheimer's Disease

Amyloid fragments, free radicals, and calcium imbalance are thought to be the pathological hallmarks of Alzheimer's disease (AD). Presenilin (PS) proteins, which are integral membrane proteins, are mainly located in the endoplasmic reticulum of neurons. Mutations in the PS genes alter proteolytic processing of the amyloid precursor protein (APP) by a gain-of-function mechanism, which is associated with the development of early-onset AD. Recently, the AD-linked PS2 mutants have been shown to influence TRPC6-enhanced Ca^2+^ entry into HEK293 cells. Transient coexpression of a loss-of-function PS2 mutant and TRPC6 enhanced angiotensin II- and 1-oleoyl-2-acetyl-sn-glycerol- (OAG-) induced Ca^2+^ entry [89]. Both A*β* and H_2_O_2_ induce death in cultured striatal cells with endogenous TRPM2, while inhibition of TRPM2 suppresses A*β*- and H_2_O_2_-induced increase in intracellular Ca^2+^ and cell death [90]. Another study shows that A*β*-induced TRPM2 currents and Ca^2+^ levels can be reduced by TRPM2 antagonism [91]. Additionally, a lack of TRPM2 rescues age-dependent spatial memory deficits in AD [92]. Therefore, these results reveal that abnormal TRPM2 activation may contribute to A*β*-related neurotoxicity and memory impairment in AD.

In APP/PS1 transgenic mice, another mouse model of AD, knockout of TRPA1 impedes AD progression, as evidenced by improved behavioral function, decreased A*β* plaque deposition, and proinflammatory cytokine production. TRPA1 antagonism induced by HC-030031 reduces astrocyte hyperactivity that is linked with A*β* production, A*β*-stimulated inflammation and astrogliosis, and CA1 neuron hyperactivity [93].

#### 3.4.2. Parkinson's Disease

Parkinson's disease (PD) is a neurodegenerative disorder that is strongly associated with the degeneration and death of dopaminergic neurons located in the substantia nigra. Using acute brain slices, functional expression of TRPM2 channels has been found in rat nigra dopaminergic neurons. Importantly, in a PD model induced by rotenone, TRPM2 activity has been observed in dopaminergic neurons, and this activation may be mediated by rotenone-induced ROS production of mitochondrion [94]. Pharmacological inhibition of TRPM2 shows an increased protection by preventing PD-linked Ca^2+^ increase and inhibited apoptosis [95].

Conversely, it has been suggested that astrocytic TRPV1 may be neuroprotective for PD. In rat models of PD, TRPV1 activity in astrocytes prevents the active degeneration of dopamine neurons and leads to behavioral recovery via endogenous production of ciliary neurotrophic factor. A similar increase is observed in human post mortem substantia nigra from PD patients by western blot and immunohistochemical analysis, implying a novel therapeutic target for PD [96]. TRPV1 activation by capsaicin can suppress spontaneous locomotion in normal rats and modulate some locomotion in reserpine-treated PD rats [97], while this suppression can be reversed by TRPV1 antagonist [98, 99].

### 3.5. Epilepsy

Epilepsy is a chronic, recurrent disorder of disturbed and synchronized electrical activity in the brain. As the important regulators of membrane potential, TRP channels contribute to neuronal depolarization, electrical activity, and firing patterns.

TRPC3 expression is highly enriched in the immature and dysplastic cortex but is only weakly expressed in a mature cortex. The combinations of low-Ca^2+^ and low-Mg^2+^ are found to induce larger depolarization in pyramidal neurons, which represents greater susceptibility to epileptiform activity in the immature and dysplastic cortex. Furthermore, TRPC3 inhibition significantly diminishes these effects, suggesting a role of enhanced TRPC3 in epileptiform activity [100]. Furthermore, TRPC3 and TRPC6 have opposite roles in neuronal death following pilocarpine-induced status epilepticus (SE). TRPC3 expression is elevated in CA1 and CA3 pyramidal cells and dentate granule cells, while TRPC6 expression is reduced in these regions. TRPC3 inhibition by Pyr3 and TRPC6 activation by hyperforin effectively protects neuronal damage due to SE [101].

Increasing evidence suggests an antiepileptic potential of TRPV1 inhibition in neuronal activity. TRPV1 antagonist capsazepine suppresses 4-aminopyridine-induced epileptiform activity *in vitro* and electrographic seizures *in vivo*, while TRPV1 agonist anandamide shows proconvulsant effects *in vivo* [102, 103]. Furthermore, this role in epilepsy is associated with the endocannabinoid system that controls neuronal excitability and regulates long-term synaptic plasticity. The levels of the endocannabinoid anandamide, a TRPV1 agonist, increase in the course of epilepsy in human neocortical brain tissue. Mice lacking the enzyme fatty acid amide hydrolase, the enzyme that hydrolyses anandamide, dramatically augment the severity of chemically induced seizures but not wild-type mice [104]. However, another cannabinoid, cannabidiol as a TRPV2 agonist, has been investigated for anticonvulsant effects. Cannabidiol is found to significantly reduce spontaneous epileptiform activity *in vitro* and incidence of severe seizures and mortality *in vivo* [105].

TRPM2 is reported to have molecular and functional interaction with EF-hand motif-containing protein (EFHC1), mutation in which causes juvenile myoclonic epilepsy (JME) via neuronal apoptosis. EFHC1 enhances TRPM2-mediated susceptibility to H_2_O_2_-induced cell death in HEK293 cells, which is reversed by JME mutations. These results suggest that TRPM2 contributes to JME phenotypes by mediating disruptive effects of EFHC1 mutations [106].

### 3.6. Mental Disorders

Schizophrenia, which is considered to be a neurodevelopmental disorder with origins in either the prenatal or neonatal period, is characterized by delusions, hallucinations, disorganized speech, and behavior. It has been reported that intrinsic somatosensory deprivation induced by neonatal capsaicin treatment, a TRPV1 activator, causes changes in the brains of rats similar to those found in schizophrenia. These changes in the rat brain and behavior suggest a possible role for TRPV1 in this neurodevelopmental disorder [107].

Various TRPs has been associated with bipolar disorders (BD), a mental disorder that causes dramatic mood shifts including emotional highs (mania or hypomania) and lows (depression). Several linkage analyses and single-nucleotide polymorphisms have shown that Trpm2 is associated with BD [108]. TRPM2-deficient mice exhibit BD-like changes in behavior and mood such as increased anxiety and decreased social responses, along with impaired electroencephalogram activity [109].

### 3.7. Pain

As an unpleasant sensory and emotional experience, pain is associated with actual or potential, tissue damage or described in terms of such damage. There is evidence suggesting that several TRP channels play a role in pain under physiological and pathological conditions. Expressed in somatosensory neurons, TRPV1 can be activated by both endogenous and exogenous stimuli including heat, arachidonic acid derivatives, vanilloids, protons, and cannabinoids. Upon activation, the pore of TRPV1 opens and allows ions for transmembrane motion to deliver a noxious message. However, TRPV1 will become rapidly desensitized upon activation, a process in which Ca^2+^ preferentially enters the cell and stimulates a series of Ca^2+^-dependent activities that ultimately lead to desensitization of the channel. This desensitization renders the channel refractory to further stimulation, leading to the paradoxical analgesic effect of TRPV1 [110].

TRPA1 is activated by pungent chemicals found in garlic, mustard, and onion. Trpa1 knockout mice display behavioral deficits in response to mustard oil, to cool and to punctate mechanical stimuli [111]. This channel is also proved to be the target through which mustard oil and garlic activate primary afferent nociceptors to produce inflammatory pain. Besides, TRPA1 can also be activated by cannabinoids to mediate currents and Ca^2+^ influx in nociceptors. However, peripheral cannabinoid compounds exert antinociceptive effects rather than analgesic effects in vivo [112]. Since TRPA1 is mostly colocalized with TRPV1 in peripheral sensory neurons, TRPV1 and TRPA1 may cross-desensitize one another when acted upon by cannabinoids or other respective agonists. An aminoalkylindole cannabinoid, WIN 55,212-2, activates TRPA1 to trigger desensitization of TRPV1 [113], whereas another TRPV1-selective cannabinoid agonist desensitizes TRPA1 [114]. These findings suggest that specific and selective modulation of TRP channel activity will be of use in alleviating pain.

## 4. Conclusions and Perspectives

A recent work in animals and humans has increased the understanding of TRPs in the brain with their widespread distribution and varying functional roles. TRP channels play important roles not only as multifunctional cellular sensors but also as modulators in growth cone guidance, synaptogenesis, spine forming, synaptic plasticity, and synaptic transmission. Additionally, the involvement of TRP channels in the pathology of numerous neurological and psychiatric disorders has been increasingly documented as important pharmacological targets. Changes in TRP expression levels or channel sensitization or desensitization have been found to be associated with the pathophysiological process and progression in TRP­related diseases. TRP activities mediated by various endogenous and exogenous agents contribute to some opposite outcomes as they exhibit different channel electrophysiological properties and operate via downstream signaling pathways. In this regard, accumulating findings highlight TRP channels as promising pharmacological targets. Therefore, further studies are required to explore the physiological and pathological roles of TRPs in the brain and develop new therapeutic strategies for the treatment of neurological and psychiatric disorders.

## Figures and Tables

**Table 1:** Evidence of proposed functions of TRPs and possible relationships to disorders in animals.

TRP channels		Evidence of expression	Proposed functions	Related disorders	References
TRPC subfamily	TRPC1	Dog brain and basilar artery	Vasospasm induced by endothelin-1 after SAH	SAH	[80]
	TRPC3	Rat cortical astrocytes	Activation and perihematomal accumulation of astrocytes; contribution to brain damage after ICH	Hemorrhagic stroke	[76, 77]
		Rat immature and dysplastic cortex; rat pyramidal cells and dentate granule cells	Low Ca^+^ and low Mg^+^-induced depolarization and epileptiform activity; contribution to neuronal death and epileptogenic insults after SE	Epilepsy	[100, 101]
	TRPC4	Rat striatal and hippocampal neurons	Upregulated expression with unknown function after ischemia	Ischemic stroke	[59]
		Dog brain and basilar artery	Vasospasm induced by endothelin-1 after SAH	SAH	[80]
	TRPC6	Rat/mouse cortical neurons	Protection against neuronal death after ischemia	Ischemic stroke	[55–58]
		Rat pyramidal cells and dentate granule cells	Inhibition of seizure susceptibility and neuronal vulnerability	Epilepsy	[101]

TRPV subfamily	TRPV1	Mouse brain	Reduced neurological and motor deficits and infarct volume in Trpv1 knockout mice after ischemia	Ischemic stroke	[70]
		Rat substantia nigra pars compacta astrocytes	Rescue of the active degeneration of dopamine neurons via endogenous production of ciliary neurotrophic factor	PD	[96, 97]
		Mouse hippocampal glia and neurons	Contribution to epileptiform activity and electrographic seizures	Epilepsy	[102, 103]
		Rat	Schizophrenia-like changes in the brain via intrinsic sensory deprivation induced by a TRPV1 activator	Schizophrenia	[107]
	TRPV4	Rat hippocampal astrocytes and neurons	Contribution to astrogliosis and neuronal death after ischemia	Ischemic stroke	[72–75]
		Rat brain	Contribution to neuronal death, brain edema, and blood-brain barrier disruption after ICH	Hemorrhagic stroke	[78]

TRPM subfamily	TRPM2	Rat microglia; mouse microglia and cortical neurons	Activation of microglia and the consequent injury and inflammation after ischemia; contribution to neuron and brain damage specifically in male after ischemia	Ischemic stroke	[61, 62]
		Rat cortex and hippocampus	Upregulated expression with unknown function after TBI; possible involvement in TBI-induced oxidative stress, apoptosis, and calcium entry	TBI	[81, 82]
		Mouse brain	Contribution to A*β*- and H_2_O_2_-induced neuronal toxicity and cell death	AD	[90]
		Rat nigral dopaminergic neurons	Changes in neuronal excitability and calcium homeostasis	PD	[94, 95]
		Mouse hippocampal neurons and ventricle cells	Contribution to the expression of JME phenotypes by mediating cell death	Epilepsy	[106]
		Mouse	BD-like changes in behavior and mood in TRPM2-deficient mice	BD	[109]
	TRPM4	Rat (ectopic expression in capillaries)	Upregulated expression in vascular endothelium to contribute to capillary death after ischemia	Ischemic stroke	[69]
			Disruption of capillary structural integrity and initiation of secondary hemorrhage after SCI	SCI	[83]
	TRPM7	Mouse brain	Contribution to excitotoxic cell death after ischemia	Ischemic stroke	[67]

TRPA subfamily	TRAP1	Mouse brain	Impediment to AD progression	AD	[93]

**Table 2:** Evidence of proposed functions of TRPs and possible relationships to neurological disorders in humans.

TRP channels		Evidence of expression	Proposed functions	Related disorders	References
TRPC subfamily	TRPC1	Human glioma cell lines	Impaired cytokinesis and facilitated glioma cell migration	Malignant gliomas	[85, 86]
	TRPC3	Human cerebral vascular tissue after hypertensive ICH	Decreased expression from hypertensive patients after intracerebral hemorrhage with unknown function	Hypertensive ICH	[79]
	TRPC1, 3, 5, 6	Human glioma cell lines	Impaired cytokinesis to become nuclear atypia and enlarged cells induced by TRPC inhibition	Malignant gliomas	[85]

TRPV subfamily	TRPV2	Human glioma cell lines	Increased chemotherapeutic drug uptake and cytotoxic activity	Glioblastoma multiforme	[87]

TRPML subfamily	TRPML2	Human glioma tissues of different levels and high-grade glioma cell lines of astrocytic origin	Increased survival and proliferation in glioma cell lines	Glioma	[88]

**Table 3:** General features and pharmacological interventions targeting TRP channels in neurological diseases.

TRP channels		Ion permeability	General physiological functions	Pharmacological interventions	References
TRPC subfamily	TRPC3	Nonselective cation	Neuronal differentiation, growth cone guidance, vasomotor	Pyr3 for stroke, epilepsy	[77, 100, 101]
	TRPC6	Nonselective cation	Axon guidance, vasomotor, smooth muscle, mechanosensor	Hyperforin for stroke, epilepsy Resveratrol for stroke OAG for AD	[55–58, 89, 101]

TRPV subfamily	TRPV1	Ca^2+^ permeable	Sensing spicy (hot) peppers, pain sensation, noxious temperature sensing	Dihydrocapsaicin for stroke Capsazepine for epilepsy, AD, PD, stroke Capsaicin for PD, schizophrenia Anandamide for epilepsy AMG9810, oleoylethanolamide for PD	[70, 71, 97–99, 102, 103, 107]
	TRPV2	Weakly Ca^2+^ selective	Thermal pain sensing, mechanosensor	Cannabidiol for glioblastoma, epilepsy	[87, 105]
	TRPV4	Ca^2+^ permeable	Osmosensing, warm sensing, nociception, pressure sensing	HC-067047 for stroke, AD, epilepsy GSK1016790A for stroke	[73, 78]

TRPM subfamily	TRPM2	Nonselective cation	Oxidant stress sensing	Tat-M2NX for stroke SB-750139 for AD 2-Aminoethoxydiphenyl borate for AD N-(p-Amylcinnamoyl) anthranilic acid for AD, PD Flufenamic acid for PD	[62, 90, 91, 95]
	TRPM4	Ca^2+^ impermeable	Mechanosensor	9-Phenanthrol for stroke	[69]
	TRPM7	Mg^2+^ permeable	Mg^2+^ homeostasis, entry pathway for trace metals	Carvacrol for stroke	[68]

TRPA subfamily	TRPA1	Nonselective cation	Pungent painful stimuli sensing, noxious cold sensing, mechanosensor	HC-030031 for AD	[93]

## Abbreviations

A*β*: Amyloid-*β*
AD: Alzheimer disease
ADPR: Adenosine diphosphoribose
APP: Amyloid precursor protein
BD: Bipolar disorders
BDNF: Brain-derived neurotrophic factor
EFHC1: EF-hand motif-containing protein
JME: Juvenile myoclonic epilepsy
LTD: Long-term depression
LTP: Long-term potentiation
mGluR: Metabotropic glutamate receptor
NMDA: N-Methyl-D-aspartate
PD: Parkinson's disease
PS: Presenilin
ROS: Reactive oxygen species
S1-6: Segments 1-6
SCI: Spinal cord injury
SE: Status epilepticus
TBI: Traumatic brain injury
tMCAO: Transient middle cerebral artery occlusion
TRP: Transient receptor potential
TRPA: Transient receptor potential ankyrin
TRPC: Transient receptor potential canonical
TRPM: Transient receptor potential melastatin
TRPML: Transient receptor potential mucolipin
TRPN: Transient receptor potential NOMPC-like
TRPP: Transient receptor potential polycystin.
